# Atypical Presentation of Duodenal Metastasis From Osteosarcoma: A Case Report

**DOI:** 10.7759/cureus.91980

**Published:** 2025-09-10

**Authors:** Maria M Pitsilka, Nikolaos Kourtides, Vasileios Zengos, Nikolaos Beis, Dimitrios Zacharoulis

**Affiliations:** 1 Department of General Surgery, University Hospital of Larissa, Larissa, GRC; 2 Department of Medicine, University Hospital of Larissa, Larissa, GRC; 3 1st Department of Surgery, Laiko General Hospital, Athens, GRC; 4 Department of Surgery, University Hospital of Larissa, Larissa, GRC

**Keywords:** diverticulum, duodenum, osteosarcoma, sarcoma, whipple

## Abstract

Α 35-year-old male, with a history of operated osteosarcoma, was hospitalized due to a computed tomography (CT) finding at the duodenum. The patient described extreme postprandial pain at the epigastrium for seven months, accompanied by weight loss. The radiologist described a thickening at the duodenum with radiopaque lesions resembling bone, dilatation at the intrahepatic bile ducts and pancreatic duct, and dislocation of the pancreas. The CT scan and gastroscopy did not have any remarkable findings. The findings of the positron emission tomography (PET)/CT​​​​showed significant hypermetabolic activity in the extensive thickening of the duodenal wall. The results necessitated the Whipple procedure.

This case highlights the need for the maintenance of high clinical suspicion, especially in patients with a history of malignancy, and the importance of diagnostic tests with greater accuracy.

## Introduction

Osteosarcoma is the most common primary bone sarcoma, with an estimated annual incidence of 800-900 patients per year in the United States and 0.3 per 100,000 people in Europe. This type of malignancy has an unusually high metastatic potential. Besides the common metastatic sites, such as lungs (in >90% of known metastatic cases, through an assumed hematogenous route) and pleura, metastases to unusual sites, such as liver, brain, and regional lymph nodes, have also been reported [1].

The primary and essential treatment for this condition is surgery, representing the sole opportunity for a cure, while preoperative radiotherapy or adjuvant chemotherapy are not to be considered as standard of care [2-4]. Complete resection may necessitate a pancreaticoduodenectomy (PD). However, due to elevated complication and recurrence rates, the decision to perform PD should be approached cautiously, and it may not be suitable for most patients [2]. Gastrointestinal metastases represent an extraordinarily rare event in the natural history of this neoplasia, and only 13 cases of osteosarcoma with metastasis at the duodenum have been reported. However, autopsy studies indicate that the rates of nonpulmonary, nonosseous metastasis in osteosarcoma may be higher than realized in clinical practice [5,6]. Unfortunately, there are no data for outcomes following their resection.

## Case presentation

Our patient is a 35-year-old Caucasian male with a history of left lower limb osteosarcoma. At this point in time, the patient presented to the hospital with severe postprandial pain at the epigastrium and weight loss of almost 15 kg over the last month.

**Figure 1:**
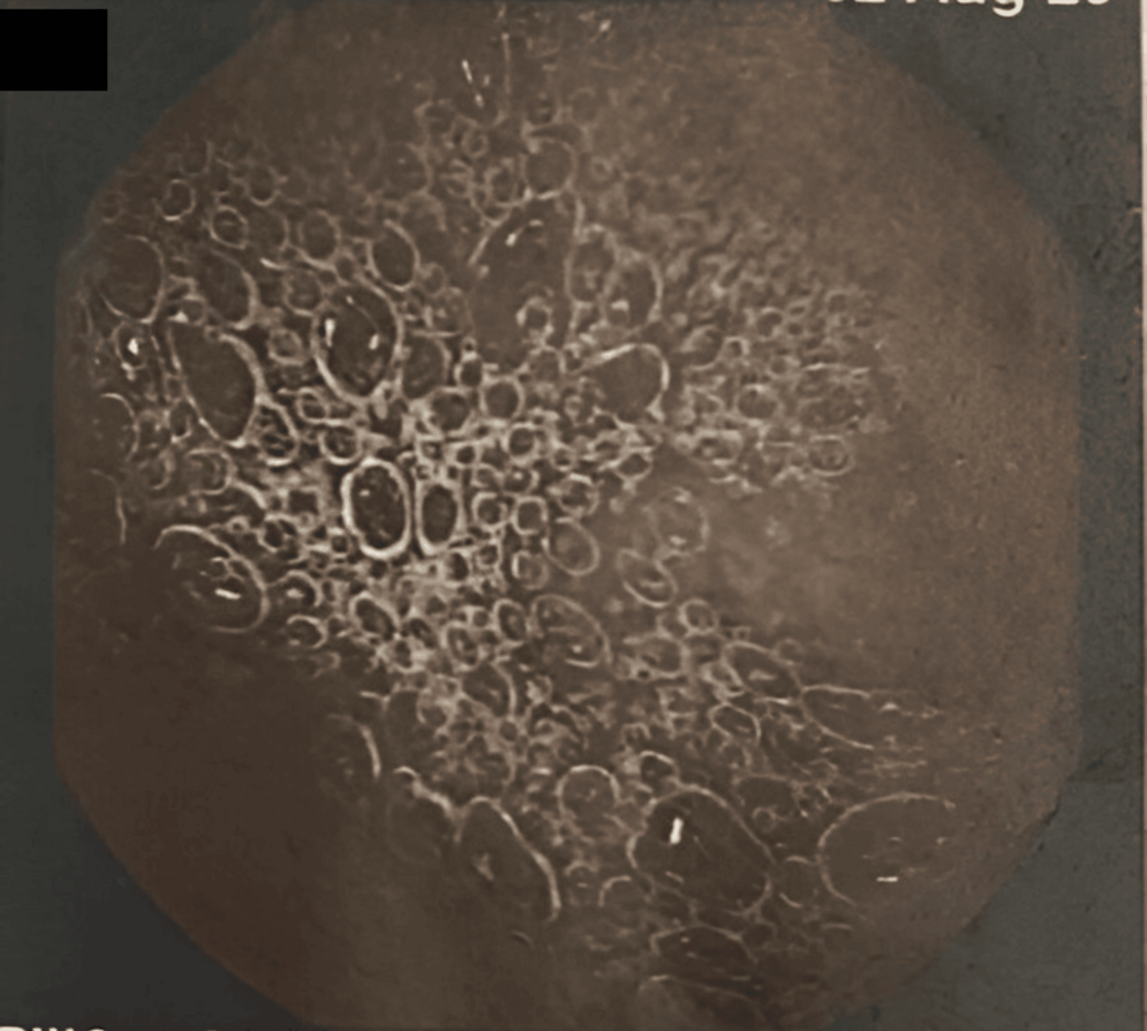
Upper GI endoscopy result – normal duodenum

**Figure 2:**
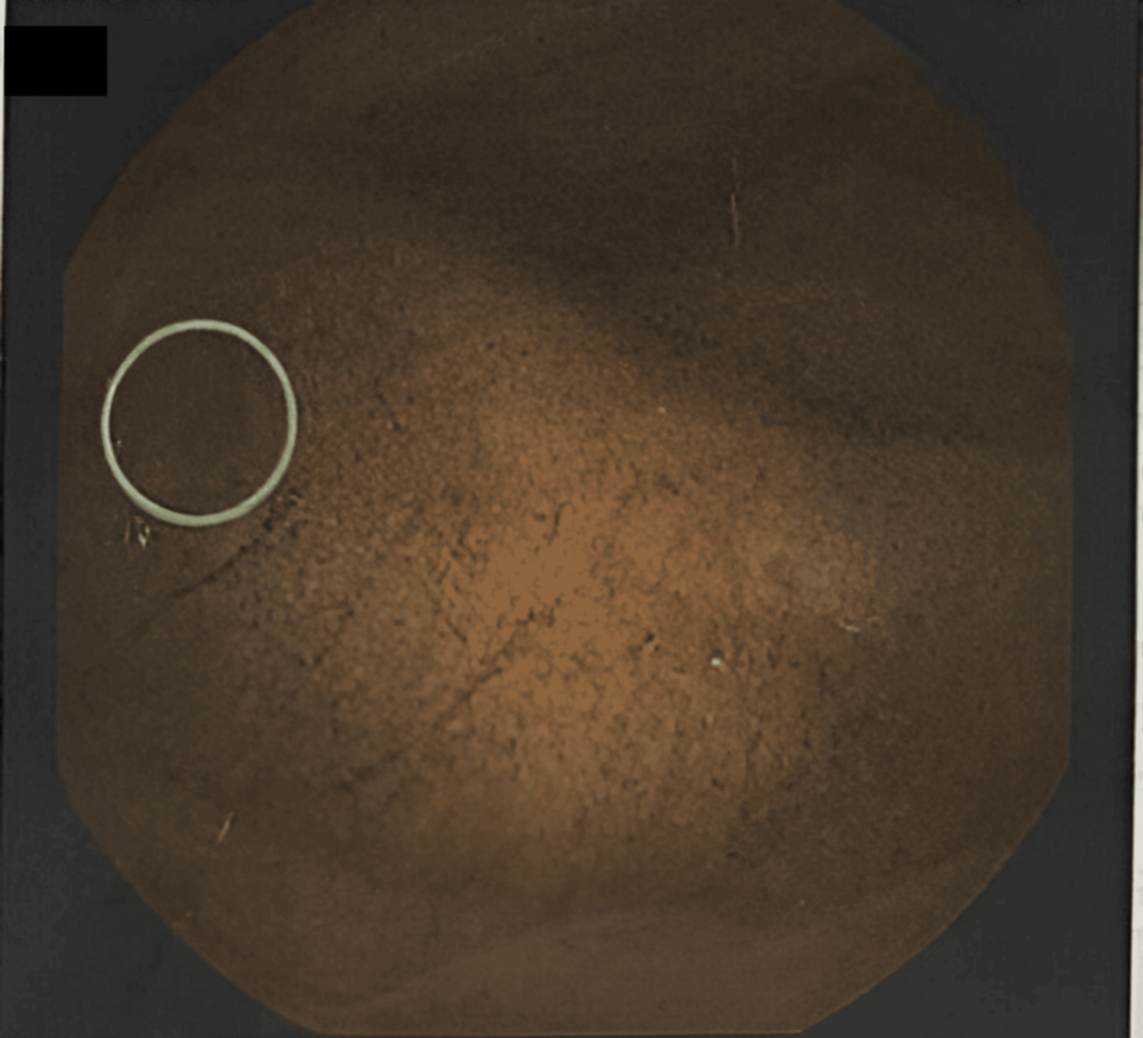
Capsule endoscopy - telangiectasia in the middle of the jejunum

Five months ago, the patient presented with melaena and mild epigastric pain. Rectal and stool examinations identified blood, suggesting an upper gastrointestinal source, while laboratory results indicated mild iron-deficiency anemia. The patient underwent an upper GI endoscopy (Figures 1, 2) in order to detect the location of the bleeding. The examination didn’t reveal any important pathological findings, except for mild gastritis.

Consequently, a colonoscopy was ordered to rule out a possible lower GI bleeding. The examination revealed no significant pathological findings up to 30 cm inside the terminal part of the ileum.

Since both the jejunum and ileum had not been thoroughly checked during the upper and lower GI endoscopies, a capsule endoscopy was performed, which revealed two small telangiectasias in the middle of the jejunum, but it was concluded that they could not be responsible for the patient’s symptoms. Therefore, the patient was diagnosed with mild gastritis, by exclusion, prescribed proton-pump inhibitors (PPIs), and discharged from the hospital.

After one month, the patient visited the hospital again with the same symptoms and an additional loss of both appetite and weight. This time, a CT scan of the upper-lower abdomen and retroperitoneum (without an IV contrast agent due to impaired renal function) was provided, but no pathological findings were revealed. The patient was once more released from the hospital without any additional medication or advice.

**Figure 3:**
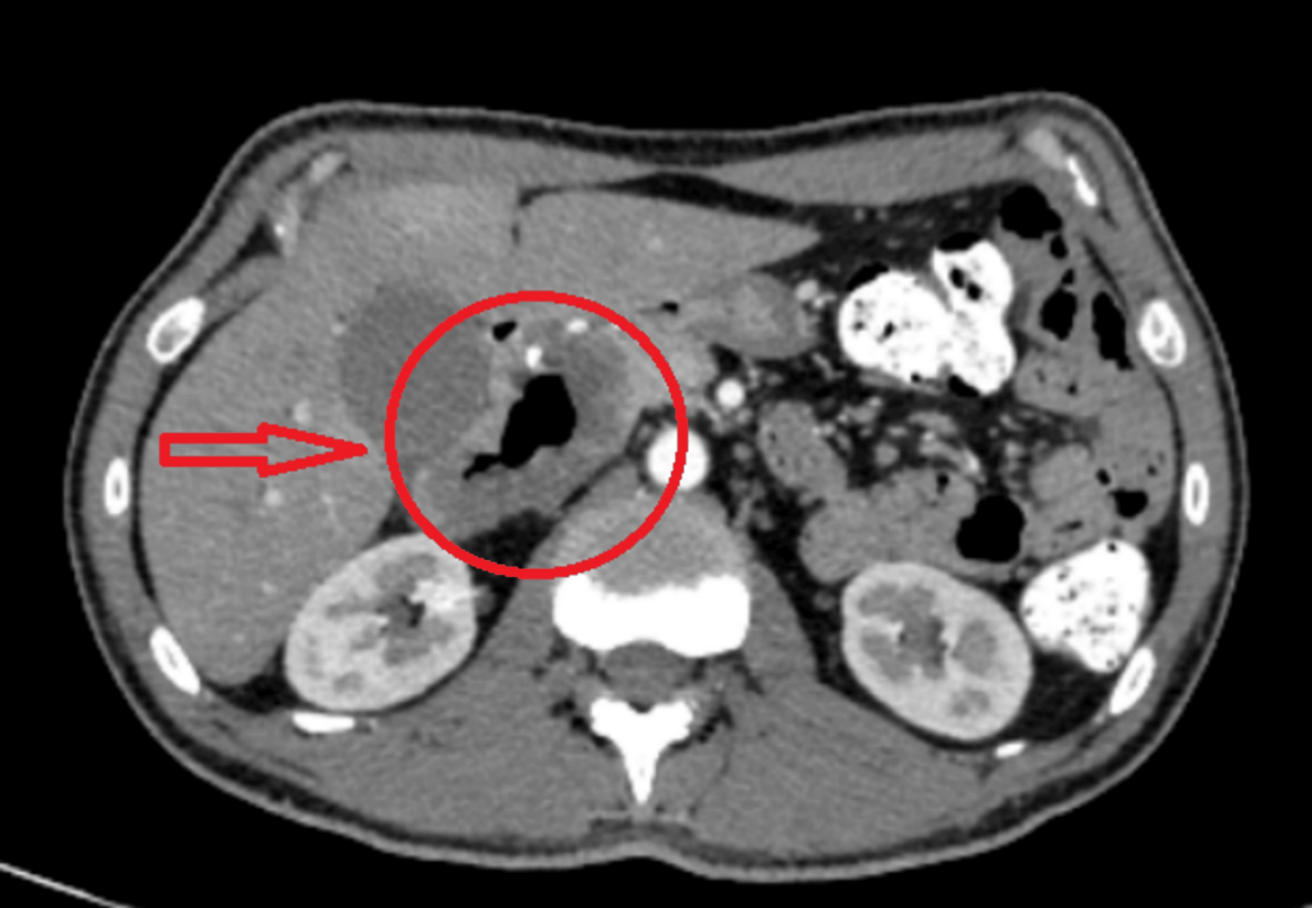
Preoperative CT scan revealing extensive thickening of the duodenal wall

More than two months later, the patient presented to the emergency department with aggravating pain in the epigastric region, which “kept him awake at night.” After a quick clinical and laboratory review (Table 1), a 4-phase CT scan was ordered(Figure 3). The results showed intrahepatic biliary dilation, a dilated common bile duct (up to 1.8 cm), and a hydrops-like gallbladder dilated up to 11 cm × 4 cm. Additionally, a diverticulum-like image with notable thickening of the wall was observed in the superior and descending parts of the duodenum (5 cm × 6 cm × 1.5 cm). Mildly dilated lymph nodes were found in the inferior vena cava and portal vein, and a repulsion of the head of the pancreas was underlined. Both the inferior and ascending parts of the duodenum were clear of any pathological findings, and a more meticulous clinical and lab examination was suggested.

**Table 1:** Preoperative laboratory values of the patient and reference values

	PATIENT	NORMAL RANGE
WBC (white blood cells)	7000	4500-10500
HGB (hemoglobin)	10.7	14-18
HCT (hematocrit)	33.5	42-52
NE% (neutrophils percentage)	73.1	40-70
LY% (lymphocytes percentage)	16,9	20-45
CRP (C-reactive protein)	0,97	<0,5
SGOT (serum glutamic-oxaloacetic transaminase)	193	<40
SGPT (serum glutamic-pyruvic transaminase)	397,2	<41
ALB (albumin)	4,79	3,5-5,2
ALP (alkaline phosphatase)	225	40-130
Amylase	47	28-100
G-GT (gamma-glutamyl transferase)	370	8,1-61
GLU (glucose)	104	74-106
K (potassium)	4,3	3,5-5,1
Na (sodium)	137,3	136-145
DBIL (direct bilirubin)	2,97	0,1-0,3
TBIL (total bilirubin)	3,4	<1,2
AFP (alpha-fetoprotein)	6,2	<7
CA-125 (cancer antigen 125)	3,2	<35
CA-15.3 (cancer antigen 15.3)	28,47	<25
CA-19.9 (cancer antigen 19.9)	12,57	<27
PSA (prostate-specific antigen)	0,837	<4,4
CEA (carcinoembryonic antigen)	0,365	<3,4

Five days later, the patient underwent a computed pancreatography that confirmed the existence of a 4 cm × 3.5 cm dilation next to the ampulla of Vater, with irregular thickening of the bowel wall up to 11.7 mm. Focal blurring of the local fat tissue and contact of the lesion with the head of the pancreas were also observed, along with a dilated gallbladder (4.5 cm × 9.5 cm) and common bile duct (1.5 cm), which was attributed to pressure exerted by the lesion. No dilation of the pancreatic duct was noted, and the pancreas seemed to absorb the contrast agent homogeneously. Due to these concerning findings, as the duodenum lesion could correspond either to a complicated diverticulum or a neoplastic proliferation, an endoscopic ultrasound and biopsy were recommended.

Two days later, an endoscopic ultrasound (EUS) took place, and although a biopsy could not be retrieved, the ultrasound findings were enlightening enough. A hemorrhagic lesion with ulceration and a visible blood vessel was found in the superior and descending duodenum, which was concluded to be the cause of the gastric obstruction. The following day, a PET scan took place, and the neoplastic nature of the proliferation was confirmed, as increased absorption of the radioactive agent (F-FDG or fludeoxyglucose F18) was highlighted in the corresponding region.

At the oncology council, the tumor was characterized as resectable; it did not penetrate large vessels, and therefore, immediate surgical treatment was preferred.

Four days later, a difficult Whipple procedure took place, and the tumor was successfully removed with clear macroscopic and microscopic resection margins. The patient stayed at the hospital for 10 days after the procedure, having a good post-operative recovery without any complications.

The surgical resection specimen, which consisted of a 7 cm × 5 cm pancreas, a 33 cm part of the small bowel, a 5 cm part of the stomach, a 14 cm × 5 cm gallbladder, and 10 lymph nodes, was sent for a histopathology review. The pathologist identified a neoplastic proliferation of 5.2 cm in the duodenum, and 3/10 lymph nodes were affected by the lesion, which was expanding by infiltrating the adjacent tissue. It was noted that both the morphology and location of the neoplasm were consistent with a gastrointestinal stromal tumor (GIST), although typical antigens characterizing a GIST were not observed [3,4]. This does not exclude the possibility of the neoplasm constituting a GIST, as in less than 2% of cases, the tumor does not have the common immunohistochemical depiction ( CD117 (KIT) and DOG1, with CD34 ) [3]. The pathologist noted that in specific areas of the neoplasm, the morphology exceeded the typical GIST features and resembled a sarcoma-like appearance with big spindle cells and adenoid formations. The pathologist noted that the possibility of this being a metastatic tumor from the osteosarcoma that the patient developed three years ago was still present. Consequently, he concluded, despite the fact that typical sarcoma agents, such as S100, SOX10, SMA, desmin, h-caldesmon, E-cadherin, EMA, DLAP, and CKAE1/AE3, were not observed, that these specific findings corroborated the presence of a mesenchymal tumor, probably resembling a high-grade sarcoma. Melanocyte markers, such as MelanA and MITF, as well as the ALK marker, which would raise suspicion of an invasive retroperitoneal carcinosarcoma, were also found to be negative.

Given the intrabdominal location of the tumor, further molecular tests were performed. A second biopsy was performed, and the immunohistochemistry revealed that the proliferation tested positive for MDM2, CDK4, ERG, SATB2, CD56, CD10, and SMA antigens. Additionally, SDHB gene expression was within normal ranges, suggesting the absence of succinate dehydrogenase (SDH)-deficient GIST, which is typically associated with mutations in this gene, while the rest of the reagents tested negative. Based on the immunohistochemical analysis and the expression of the SATB2 and MDM2 genes, we strongly support the claim that the neoplastic region is metastatic, originating from the patient’s antecedent osteosarcoma, without entirely excluding the possibility of a primary dedifferentiated liposarcoma.

## Discussion

Osteosarcoma is a highly aggressive bone malignancy known for its propensity to metastasize, primarily to the lungs and, less frequently, to soft tissues and other organs [7,8]. Visceral organ involvement is unusual, and GI metastases account for less than 1% of all cases. When they do occur, they are typically associated with widespread disease and portend a poor prognosis. The duodenum is an uncommon site even among reported GI metastases, with only isolated case reports documenting such involvement. This is one of the few reported cases in the literature of metastatic osteosarcoma in the duodenum [9-14]. Proposed mechanisms include hematogenous spread or direct invasion in cases of extensive retroperitoneal disease.

The present case highlights the challenges in both clinical and histological diagnosis and management. The patient’s initial clinical presentation, characterized by postprandial epigastric pain and weight loss, was nonspecific and did not immediately suggest metastatic disease. Imaging studies revealed duodenal thickening with radiopaque lesions resembling bone, which raised suspicion for metastatic involvement. PET/CT confirmed significant hypermetabolic activity, warranting further investigation. Prior gastroscopy and imaging had failed to detect abnormalities, illustrating the limitations of routine diagnostic modalities in identifying rare metastatic sites.

Although immunohistochemical markers were negative for typical osteosarcoma profiles, the diagnosis was supported by clinical history, radiologic evidence, and the expression of genes such as SATB2 and MDM2. This case underscores the complexity of histological diagnosis in rare metastatic sites, where typical markers may be absent, and highlights the need for a multidisciplinary approach.

According to the literature, the greatest dimension among previously reported metastatic osteosarcomas was 6.0 cm, while ours is 5.2 cm. Also, just like our case, these metastatic lesions typically present a few years after the primary diagnosis.

Surgical resection, specifically pancreaticoduodenectomy (Whipple procedure), remains the primary treatment option when complete resection is feasible [2]. However, this procedure carries considerable morbidity and should be carefully considered based on the patient's condition and tumor burden. In this case, surgical intervention was necessary given the obstructive nature of the duodenal lesion and the patient’s persistent symptoms. Although chemotherapy plays a crucial role in systemic disease control, its effectiveness in rare metastatic presentations, such as duodenal involvement, remains uncertain [9]. Widespread metastases at presentation herald a universally poor prognosis, but isolated lesions can be resected with good results. Clinicians responsible for patients with osteosarcoma should be aware of the potential for disease-free survival following resection of an isolated bowel metastasis.

## Conclusions

In conclusion, metastatic osteosarcoma of the duodenum is rare and easily misdiagnosed. It is something that clinicians should acknowledge when forming a differential diagnosis in a patient with abdominal pain and weight loss, especially when there is a history of osteosarcoma, as there are a few cases in the literature that describe it. This tumor represents a management challenge because of the lack of data in the literature. Early radical surgery is the key to better clinical outcomes.
